# Synthesis and crystal structure of 2-(benzo[*d*]thia­zol-2-yl)-*N*′-[(*E*)-1-(4-bromo­phen­yl)ethyl­idene]acetohydrazide

**DOI:** 10.1107/S2056989026001313

**Published:** 2026-02-24

**Authors:** Heba A. Elboshi, Rasha A. Azzam, Galal H. Elgemeie, Peter G. Jones

**Affiliations:** a Chemistry Department, Faculty of Science, Capital University, Helwan, Egypt; b Institut für Anorganische und Analytische Chemie, Technische Universität Braunschweig, Hagenring 30, D-38106 Braunschweig, Germany; Universität Greifswald, Germany

**Keywords:** crystal structure, benzo­thia­zole, hydrazide, hydrogen bond, halogen bond

## Abstract

The title compound (*E*)-2-(benzo[*d*]thia­zol-2-yl)-*N*′-(1-(4-bromo­phen­yl)ethyl­idene)acetohydrazide, C_17_H_14_BrN_3_OS, crystallizes in space group *P*2_1_/*c* with *Z* = 4. The configuration across the formal N=C double bond at the hydrazide moiety is *E*; the atom sequence C—C—C(= O)—N—N=C—(bromo­phen­yl) is very approximately planar (r.m.s. deviation 0.17 Å, when all carbon atoms of the bromo­phenyl group are included), being synperiplanar around the C(=O)—N bond and anti­periplanar (choosing the appropriate final atom C_Ar_) elsewhere. The inter­planar angle to the benzo­thia­zole unit (r.m.s. deviation 0.01 Å) is 69.75 (2)°. The main packing feature is a classical inversion-symmetric dimer with hydrogen bonds of the type N—H⋯O=C. This combines with a rather long N_thia­zole_⋯Br halogen bond to form a thick layer structure parallel to the *bc* plane.

## Abstract 2

The configuration across the formal N=C double bond of the 2-substituent is *E*; the atom sequence C—C—C(=O)—N—N=C—(bromo­phen­yl) of the 2-substituent is very approximately planar. A classical hydrogen bond and a halogen bond combine to form a layer structure.

## 1. Chemical context

The benzo­thia­zole scaffold is established as one of the most significant moieties in medicinal chemistry, because benzo­thia­zole derivatives are present in a broad range of natural products and bioactive compounds, and many derivatives exhibit significant activity while causing few side-effects (Keri *et al.*, 2015 ▸). Benzo­thia­zole derivatives have attracted appreciable recent attention in medicinal chemistry because of their biological and pharmacological characteristics (Gill *et al.*, 2015 ▸). Numerous biological activities, including anti­cancer, anti­fungal and anti­bacterial effects, are associated with the benzo­thia­zole moiety; a more extensive description can be found in our previous publication (Elboshi *et al.*, 2026 ▸) and references therein.

We have synthesised new heterocyclic compounds with incorporated benzo­thia­zole motifs, and these too have demonstrated noteworthy biological activity (Azzam *et al.*, 2017 ▸), *e.g.* benzo­thia­zole-substituted coumarin residues, which also have useful optical characteristics (Abdallah *et al.*, 2023 ▸). We have described some new coumarin compounds that are currently being used as laser dyes for medicinal applications (Elgemeie, 1989 ▸). We have also reported new benzo­thia­zole-based heterocycles that showed significant fluorescence as well as biological importance (Azzam *et al.*, 2022 ▸).

**Figure 1:**
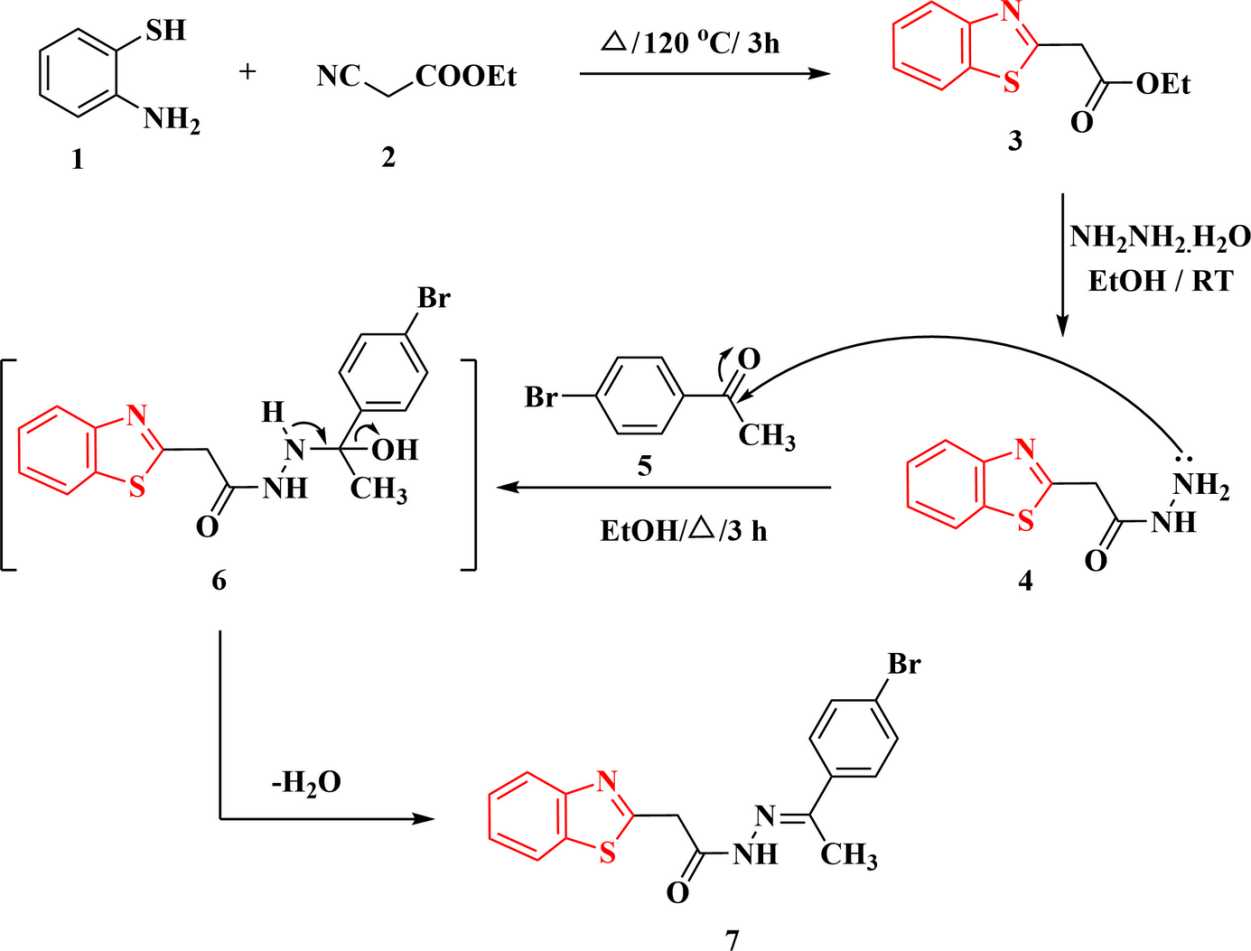
The synthesis of compound **7**.

The goal of the current study was to design and produce benzo­thia­zolyl ethyl­idene-acetohydrazides inspired by the results of our earlier work. The 2-(benzo­thia­zol­yl)-[1-(4-bromo­phen­yl)ethyl­idene]acetohydrazide derivative **7** was synthesized in good yield by reacting 2-benzo­thia­zolyl aceto­hydrazide **4** with 4-bromo­phenyl­aceto­phenone **5** in refluxing ethanol for 3 h (Fig. 1 ▸). The crystal structure of **7** was determined and is reported here.
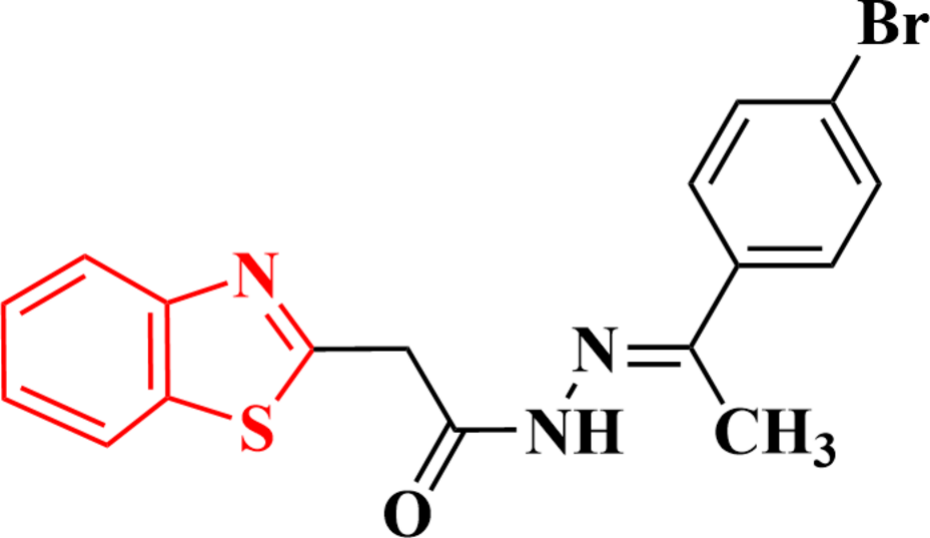


## 2. Structural commentary

**Figure 2:**
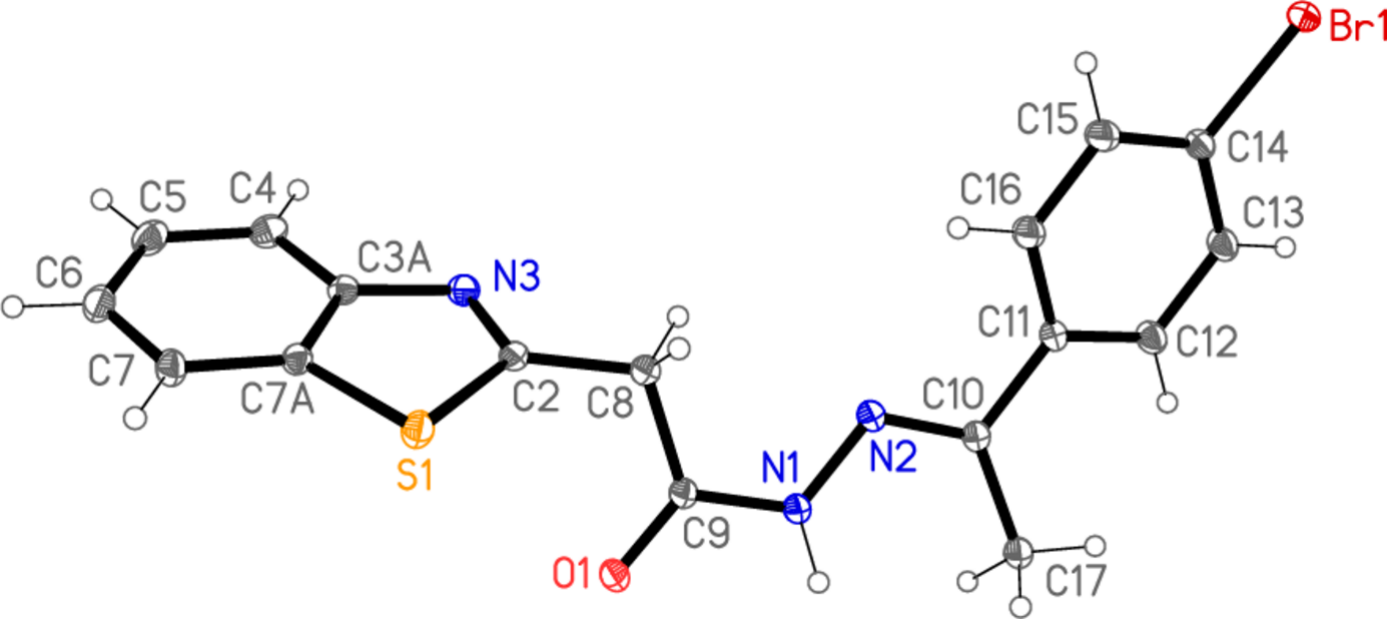
The mol­ecule of compound **7** in the crystal. Ellipsoids correspond to 50% probability levels.

**Figure 3:**
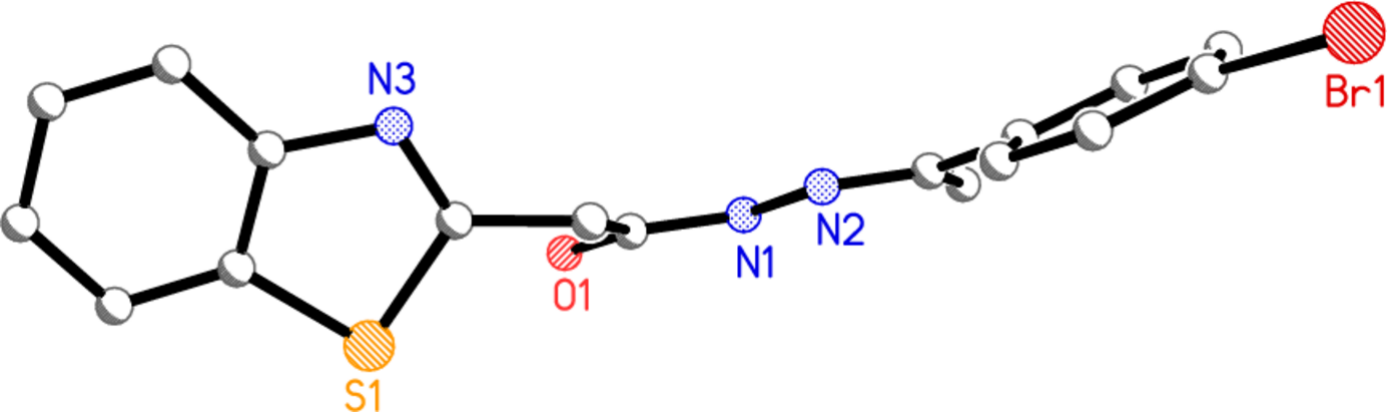
‘Side view’ of the mol­ecule of **7**; hydrogen atoms are omitted and radii are arbitrary.

The mol­ecule of compound **7** is shown in Fig. 2 ▸, with selected geometric parameters in Table 1 ▸. The mol­ecular dimensions may be regarded as normal, *e.g.* the wide exocyclic angles at C3*A* and C7*A*. The configuration across the formal double bond N2=C10 is *E*, and this bond is some 0.06 Å shorter than the formal single bond N1—C9, although formal bond orders should be inter­preted carefully in view of probable delocalization of the multiple bonding. The approximate synperi­planarity of the sequence C2—C8—C9—O1 is associated with the intra­molecular S1⋯O1 contact of 3.1729 (10) Å, a feature that we have frequently observed in related compounds, generally with much shorter contact distances (*e.g.* Elboshi *et al.*, 2026 ▸). As can be seen from the side view of the mol­ecule in Fig. 3 ▸, the atom sequence C2–C8–C9–N1–N2–C10–C11–C12 is very approximately planar (r.m.s. deviation 0.17 Å, including the carbon atoms of the bromo­phenyl group), being synperi­planar around the bond C9—N1 and anti­periplanar elsewhere. The inter­planar angle to the plane of the benzo­thia­zole unit (r.m.s. deviation 0.01 Å) is 69.75 (2)°. The coordination geometry at the nitro­gen atom N1 of the NH group is planar (r.m.s. deviation of 4 atoms 0.003 Å).

## 3. Supra­molecular features

**Figure 4:**
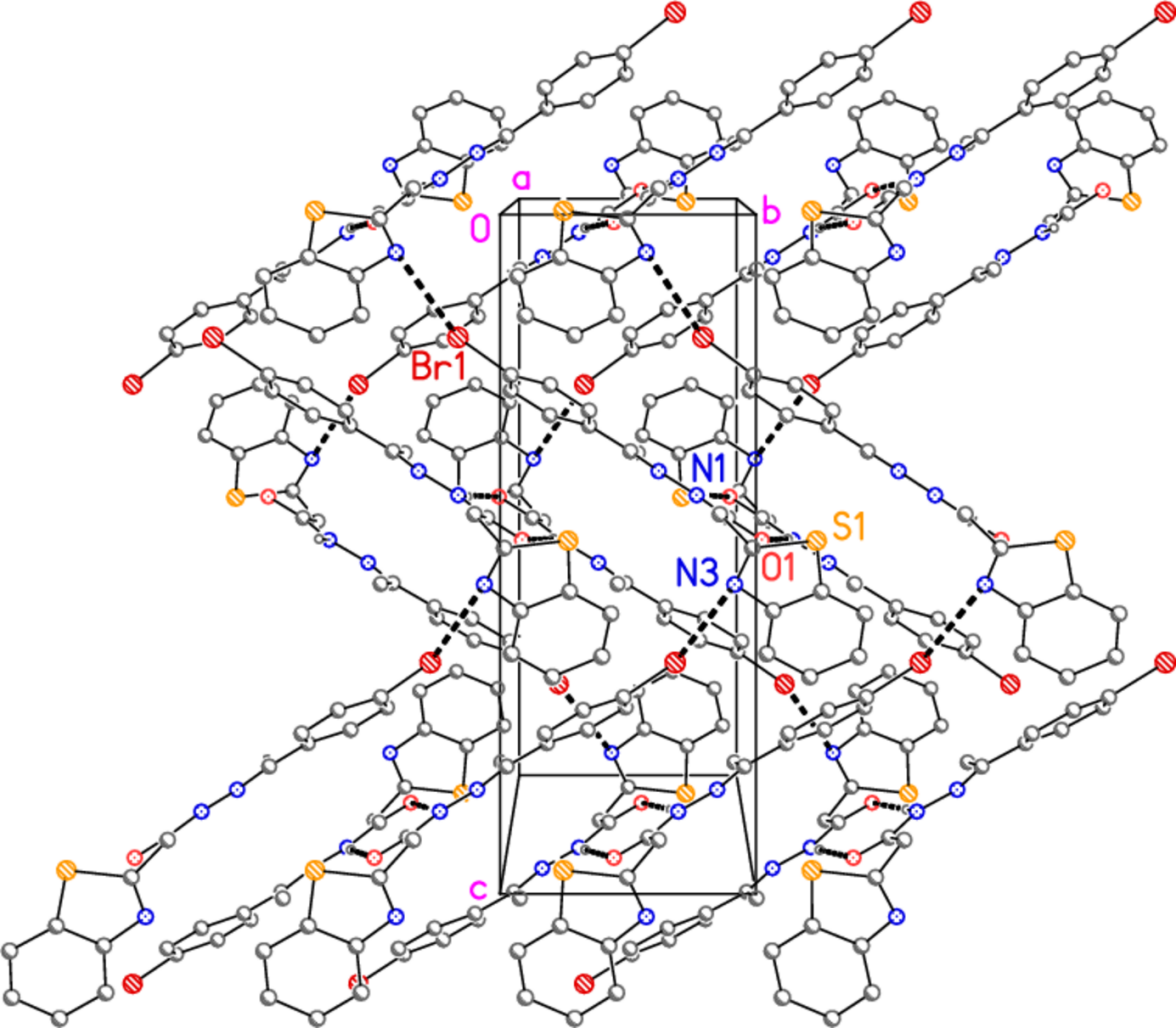
Packing diagram of compound **7**: One layer viewed perpendicular to the *bc* plane, showing classical hydrogen bonds (seen almost end-on, *cf*. Fig. 5 ▸) and halogen bonds (both as thick dashed lines). Hydrogen atoms not involved in the hydrogen bonding are omitted. Labels indicate atoms of the asymmetric unit.

**Figure 5:**
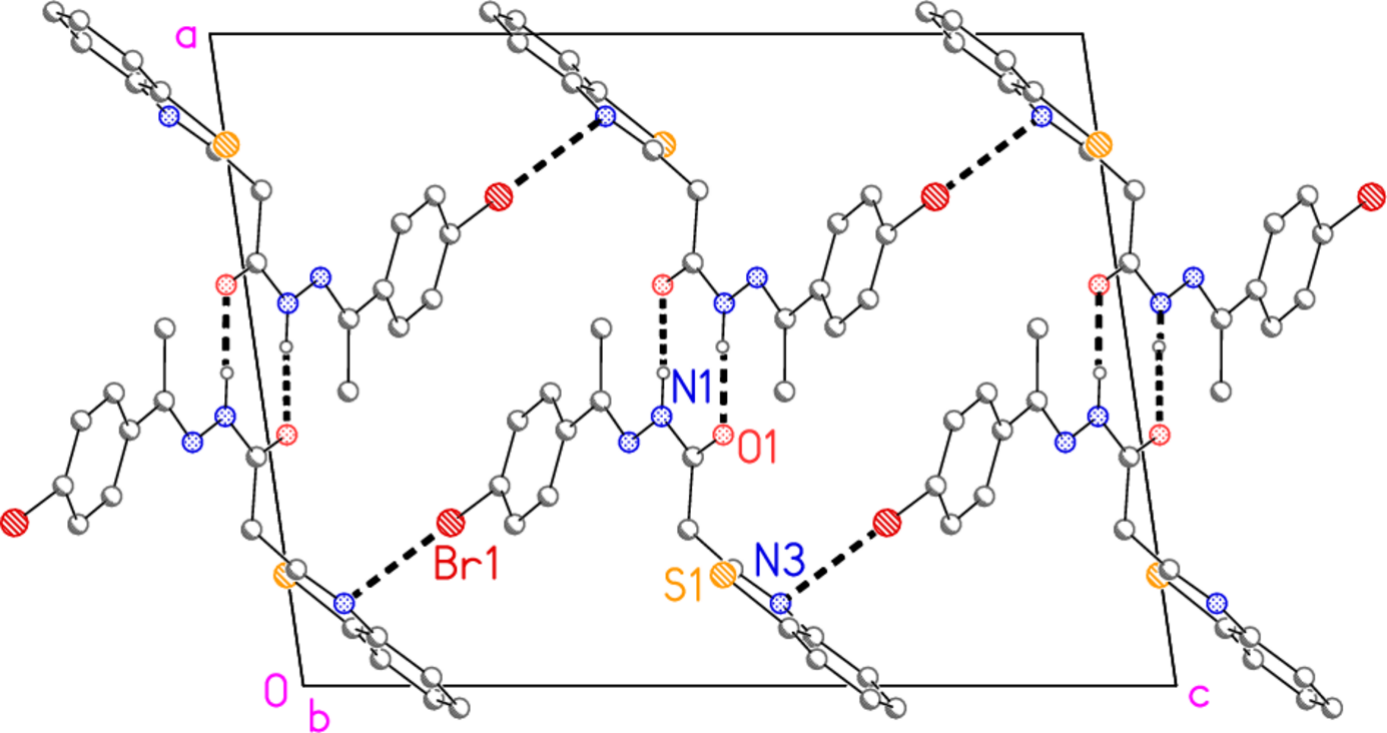
Simplified packing diagram of compound **7** shown as a projection parallel to the *b* axis.

The NH group at N1 is involved in a classical hydrogen bond to O1 via an inversion centre (Table 2 ▸), leading to the well-known motif with graph set 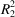
(8). Three borderline ‘weak’ hydrogen bonds are also included in Table 2 ▸. There is a further contact N3⋯Br1 (*x*, 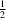 − *y*, 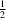 + *z*) of 3.1572 (10) Å, with an N⋯Br—C angle of 161.26 (4)°, that may be regarded as a ‘halogen bond’ [for reviews of this topic, see e.g. Cavallo *et al.* (2016 ▸) or Metrangolo *et al.* (2008 ▸)]. The combination of classical hydrogen bond plus halogen bond leads to the formation of a layer structure (Fig. 4 ▸) parallel to the *bc* plane and with a thickness equal to the length of the *a* axis. Because the hydrogen bonds are seen almost end-on in Fig. 4 ▸, a projection of the structure parallel to the *b* axis (Fig. 5 ▸) is also shown for the sake of clarity.

## 4. Database survey

Searches were conducted using CSD Version 6.00 (update August 2025; Groom *et al.*, 2016 ▸) and the ConQuest routine (Bruno *et al.*, 2002 ▸), Version 2025.2.0. The main search was based on the standard benzo[*d*]thia­zole ring system with appropriately defined coordination numbers but no limitations on bond orders or on substituents except for that at C2; this substituent was set to –C^4^–C^3^(–O^1^)–N^any^ (as in **7**), where the superscripts indicate coordination number, whereby all bond orders in this fragment were allowed. This gave 18 hits. Only three of these structures had a 2-substituent of the type –C^4^–C^3^(–O^1^)–N^any^–N^any^ (as in **7**): 4-(1,3-benzo­thia­zol-2-yl)-5-methyl-2-phenyl-4-(prop-2-en-1-yl)-2,4-di­hydro-3*H*-pyrazol-3-one (refcode DOMYAI: Chakib *et al.*, 2019 ▸) and our previous structures, the hydrazine derivatives *N*′-[(1,3-benzo­thia­zol-2-yl)acet­yl]benzohydrazide (IYUSIH; Azzam *et al.*, 2021 ▸) and 2-(1,3-benzo­thia­zol-2-yl)-*N*′-[(4-methyl­phen­yl)sulfon­yl]acetohydrazide (JEBQOZ; Azzam *et al.*, 2017 ▸). Similarly, only three structures involved the 2-substituent –CH_2_–C^3^(–O^1^)–N^any^ (as in **7**), namely IYUSIH, JEBQOZ and 2-(1,3-benzo­thia­zol-2-yl)-*N*-(2-hy­droxy­phen­yl)acetamide (HANREW; Dauer *et al.*, 2017 ▸).

## 5. Synthesis and crystallization

A mixture of 2-benzo­thia­zolyl acetohydrazide **4** (2.072 g, 0.01 mol) and 4-bromo­phenyl­aceto­phenone **5** (3.98 g, 0.02 mol) was refluxed in ethanol (30 mL) for 3 h. The colourless solid product **7** thus formed was filtered from the hot solution, washed with a mixture of petroleum ether and ethyl acetate (1:1) and then recrystallized from ethanol.

Yellow solid; yield 80%; m.p. 466–468 K. IR (KBr, cm^−1^): *ν* 3182 (NH), 3054 (Ar—CH), 1669 (CO); ^1^H NMR (400 MHz, DMSO-*d_6_*): δ 2.22 (*s*, 3H, CH_3_), 4.36 (*s*, 2H, CH_2_), 7.38–7.62 (*m*, 4H, Ar-H & benzo­thia­zole-H), 7.74 (*d*, *J* = 8.4, 2H, Ar-H), 7.96 (*d*, *J* = 8.0 Hz, 1H, benzo­thia­zole-H), 8.04 (*d*, *J* = 7.6 Hz, 1H, benzo­thia­zole-H), 10.90 (*s*, 1H, NH). Analysis: calculated for C_17_H_14_BrN_3_OS (388.28): C 52.59, H 3.63, N 10.82. Found: C 52.53, H 3.60, N 10.81%.

## 6. Refinement

Details of data collection and structure refinement are summarized in Table 3 ▸. The benzo­thia­zole system was assigned the standard IUPAC numbering. The hydrogen atom of the NH group was refined freely. The methyl group was refined as an idealized rigid group with C—H = 0.98 Å, H—C—H = 109.5°, allowed to rotate but not tip (AFIX 137). Other hydrogen atoms were included using a riding model starting from calculated positions (C—H_arom_ = 0.95, C—H_methyl­ene_ = 0.98 Å). The *U*(H) values were fixed at 1.5 × *U*_eq_ of the parent carbon atoms for the methyl group and 1.2 × *U*_eq_ for the other hydrogens.

## Supplementary Material

Crystal structure: contains datablock(s) I, global. DOI: 10.1107/S2056989026001313/yz2073sup1.cif

Structure factors: contains datablock(s) I. DOI: 10.1107/S2056989026001313/yz2073Isup2.hkl

Supporting information file. DOI: 10.1107/S2056989026001313/yz2073Isup3.cml

CCDC reference: 2529228

Additional supporting information:  crystallographic information; 3D view; checkCIF report

## Figures and Tables

**Table 1:** Selected geometric parameters (Å, °)

S1—C7*A*	1.7342 (12)	C9—O1	1.2307 (15)
S1—C2	1.7507 (12)	C9—N1	1.3543 (15)
C2—N3	1.2976 (16)	N1—N2	1.3764 (15)
N3—C3*A*	1.3909 (16)	N2—C10	1.2942 (15)
C3*A*—C7*A*	1.4090 (17)		
			
C7*A*—S1—C2	89.24 (6)	C7—C7*A*—S1	129.27 (10)
N3—C2—S1	115.90 (9)	C3*A*—C7*A*—S1	109.07 (8)
C2—N3—C3*A*	110.63 (10)	C9—N1—N2	119.49 (10)
N3—C3*A*—C4	125.01 (11)	C10—N2—N1	116.53 (10)
N3—C3*A*—C7*A*	115.16 (10)		
			
N3—C2—C8—C9	−115.73 (13)	C9—N1—N2—C10	−179.12 (12)
C2—C8—C9—O1	−10.43 (19)	N1—N2—C10—C11	179.74 (11)
C2—C8—C9—N1	170.32 (12)	N2—C10—C11—C12	−160.60 (12)
C8—C9—N1—N2	−1.76 (18)		

**Table 2:** Hydrogen-bond geometry (Å, °)

*D*—H⋯*A*	*D*—H	H⋯*A*	*D*⋯*A*	*D*—H⋯*A*
N1—H01⋯O1^i^	0.91 (2)	1.98 (2)	2.8839 (14)	168 (2)
C7—H7⋯N3^ii^	0.95	2.68	3.5942 (17)	161
C17—H11*B*⋯Br1^iii^	0.98	2.93	3.7666 (12)	145
C17—H11*C*⋯N2^iv^	0.98	2.69	3.5464 (18)	147

Symmetry codes: (i) 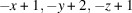; (ii) 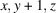; (iii) 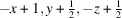; (iv) 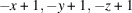.

**Table 3:** Experimental details

Crystal data	
Chemical formula	C_17_H_14_BrN_3_OS
*M*_r_	388.28
Crystal system, space group	Monoclinic, *P*2_1_/*c*
Temperature (K)	100
*a*, *b*, *c* (Å)	13.4768 (3), 6.73234 (16), 17.8734 (5)
β (°)	98.169 (2)
*V* (Å^3^)	1605.21 (7)
*Z*	4
Radiation type	Mo *K*α
μ (mm^−1^)	2.70
Crystal size (mm)	0.25 × 0.10 × 0.04

Data collection	
Diffractometer	XtaLAB Synergy
Absorption correction	Multi-scan (*CrysAlis PRO*; Rigaku OD, 2024 ▸)
*T*_min_, *T*_max_	0.563, 1.000
No. of measured, independent and observed [*I* > 2σ(*I*)] reflections	143532, 8606, 7894
*R*_int_	0.063
θ values (°)	θ_max_ = 37.8, θ_min_ = 2.3
(sin θ/λ)_max_ (Å^−1^)	0.862

Refinement	
*R*[*F*^2^ > 2σ(*F*^2^)], *wR*(*F*^2^), *S*	0.047, 0.085, 1.18
No. of reflections	8606
No. of parameters	213
H-atom treatment	H atoms treated by a mixture of independent and constrained refinement
Δρ_max_, Δρ_min_ (e Å^−3^)	1.44, −0.71

Computer programs: *CrysAlis PRO* (Rigaku OD, 2024 ▸), *SHELXT* (Sheldrick, 2015*a* ▸), *SHELXL2019/3* (Sheldrick, 2015*b* ▸), *XP* (Bruker, 1998 ▸) and *publCIF* (Westrip, 2010 ▸).

## supplementary crystallographic information

### 2-(Benzo[d]thiazol-2-yl)-N'-[(E)-1-(4-bromophenyl)ethylidene]acetohydrazide Crystal data

**Table:**

C_17_H_14_BrN_3_OS	*F*(000) = 784
*M**_r_* = 388.28	*D*_x_ = 1.607 Mg m^−^^3^
Monoclinic, *P*2_1_/*c*	Mo *K*α radiation, λ = 0.71073 Å
*a* = 13.4768 (3) Å	Cell parameters from 56121 reflections
*b* = 6.73234 (16) Å	θ = 2.3–43.5°
*c* = 17.8734 (5) Å	µ = 2.70 mm^−^^1^
β = 98.169 (2)°	*T* = 100 K
*V* = 1605.21 (7) Å^3^	Lath, colourless
*Z* = 4	0.25 × 0.10 × 0.04 mm

### 2-(Benzo[d]thiazol-2-yl)-N'-[(E)-1-(4-bromophenyl)ethylidene]acetohydrazide Data collection

**Table:**

XtaLAB Synergy diffractometer	8606 independent reflections
Radiation source: micro-focus sealed X-ray tube, PhotonJet (Mo) X-ray Source	7894 reflections with *I* > 2σ(*I*)
Mirror monochromator	*R*_int_ = 0.063
Detector resolution: 10.0000 pixels mm^-1^	θ_max_ = 37.8°, θ_min_ = 2.3°
ω scans	*h* = −23→23
Absorption correction: multi-scan (CrysAlisPro; Rigaku OD, 2024)	*k* = −11→11
*T*_min_ = 0.563, *T*_max_ = 1.000	*l* = −30→30
143532 measured reflections	

### 2-(Benzo[d]thiazol-2-yl)-N'-[(E)-1-(4-bromophenyl)ethylidene]acetohydrazide Refinement

**Table:**

Refinement on *F*^2^	Primary atom site location: dual
Least-squares matrix: full	Hydrogen site location: mixed
*R*[*F*^2^ > 2σ(*F*^2^)] = 0.047	H atoms treated by a mixture of independent and constrained refinement
*wR*(*F*^2^) = 0.085	*w* = 1/[σ^2^(*F*_o_^2^) + (0.0311*P*)^2^ + 1.1712*P*] where *P* = (*F*_o_^2^ + 2*F*_c_^2^)/3
*S* = 1.18	(Δ/σ)_max_ = 0.001
8606 reflections	Δρ_max_ = 1.44 e Å^−^^3^
213 parameters	Δρ_min_ = −0.71 e Å^−^^3^
0 restraints	

### 2-(Benzo[d]thiazol-2-yl)-N'-[(E)-1-(4-bromophenyl)ethylidene]acetohydrazide Fractional atomic coordinates and isotropic or equivalent isotropic displacement parameters (Å^2^)

**Table:**

	*x*	*y*	*z*	*U*_iso_*/*U*_eq_	
S1	0.16962 (2)	1.25918 (5)	0.49911 (2)	0.01614 (6)	
C2	0.17813 (8)	1.00142 (18)	0.51133 (7)	0.01334 (18)	
N3	0.12596 (8)	0.92656 (15)	0.56014 (6)	0.01357 (16)	
C3A	0.07440 (8)	1.07504 (17)	0.59266 (7)	0.01226 (17)	
C4	0.01259 (9)	1.0444 (2)	0.64826 (7)	0.0167 (2)	
H4	0.003219	0.914827	0.667136	0.020*	
C5	−0.03465 (10)	1.2067 (2)	0.67519 (8)	0.0185 (2)	
H5	−0.076718	1.188063	0.712990	0.022*	
C6	−0.02120 (9)	1.3985 (2)	0.64740 (8)	0.0176 (2)	
H6	−0.055025	1.507238	0.666381	0.021*	
C7	0.04045 (9)	1.43262 (19)	0.59281 (7)	0.0156 (2)	
H7	0.049896	1.562658	0.574429	0.019*	
C7A	0.08812 (8)	1.26853 (17)	0.56588 (7)	0.01244 (17)	
C8	0.23962 (9)	0.8749 (2)	0.46650 (8)	0.0168 (2)	
H8A	0.217578	0.898276	0.411983	0.020*	
H8B	0.227229	0.733355	0.477020	0.020*	
C9	0.35099 (8)	0.91582 (18)	0.48404 (7)	0.01371 (18)	
O1	0.38489 (7)	1.05873 (16)	0.52236 (7)	0.0214 (2)	
N1	0.41315 (8)	0.78849 (16)	0.45479 (7)	0.01472 (17)	
H01	0.4799 (18)	0.818 (3)	0.4639 (13)	0.028 (6)*	
N2	0.37355 (8)	0.62797 (16)	0.41312 (6)	0.01411 (17)	
C10	0.43690 (9)	0.51041 (18)	0.38756 (7)	0.01316 (18)	
C11	0.39222 (9)	0.33802 (17)	0.34310 (7)	0.01298 (18)	
C12	0.44925 (9)	0.17000 (18)	0.33140 (7)	0.01525 (19)	
H13	0.517977	0.165244	0.352572	0.018*	
C13	0.40666 (9)	0.00945 (19)	0.28910 (7)	0.0158 (2)	
H14	0.445809	−0.104428	0.281831	0.019*	
C14	0.30657 (9)	0.01796 (18)	0.25778 (7)	0.01412 (18)	
Br1	0.24979 (2)	−0.19335 (2)	0.19566 (2)	0.01864 (4)	
C15	0.24758 (9)	0.1824 (2)	0.26853 (8)	0.0181 (2)	
H16	0.179047	0.186459	0.246826	0.022*	
C16	0.29056 (9)	0.34051 (19)	0.31154 (8)	0.0171 (2)	
H17	0.250470	0.452312	0.319744	0.021*	
C17	0.54844 (9)	0.5433 (2)	0.40014 (8)	0.0166 (2)	
H11A	0.564414	0.664090	0.373666	0.025*	
H11B	0.582002	0.429331	0.380591	0.025*	
H11C	0.571579	0.557948	0.454370	0.025*	

### 2-(Benzo[d]thiazol-2-yl)-N'-[(E)-1-(4-bromophenyl)ethylidene]acetohydrazide Atomic displacement parameters (Å^2^)

**Table:**

	*U*^11^	*U*^22^	*U*^33^	*U*^12^	*U*^13^	*U*^23^
S1	0.01733 (12)	0.01212 (11)	0.02066 (14)	0.00074 (9)	0.00852 (10)	0.00166 (10)
C2	0.0104 (4)	0.0123 (4)	0.0171 (5)	0.0004 (3)	0.0012 (3)	−0.0023 (4)
N3	0.0124 (4)	0.0112 (4)	0.0167 (4)	0.0010 (3)	0.0007 (3)	0.0009 (3)
C3A	0.0112 (4)	0.0119 (4)	0.0133 (4)	0.0005 (3)	0.0005 (3)	0.0013 (3)
C4	0.0156 (4)	0.0188 (5)	0.0161 (5)	−0.0014 (4)	0.0032 (4)	0.0030 (4)
C5	0.0149 (4)	0.0250 (6)	0.0162 (5)	−0.0006 (4)	0.0048 (4)	−0.0010 (5)
C6	0.0142 (4)	0.0208 (5)	0.0178 (5)	0.0029 (4)	0.0025 (4)	−0.0036 (4)
C7	0.0155 (4)	0.0131 (4)	0.0181 (5)	0.0030 (4)	0.0022 (4)	−0.0011 (4)
C7A	0.0116 (4)	0.0113 (4)	0.0145 (5)	0.0010 (3)	0.0022 (3)	0.0005 (3)
C8	0.0114 (4)	0.0165 (5)	0.0226 (6)	−0.0001 (4)	0.0021 (4)	−0.0074 (4)
C9	0.0111 (4)	0.0137 (4)	0.0163 (5)	0.0006 (3)	0.0018 (3)	−0.0037 (4)
O1	0.0125 (3)	0.0209 (4)	0.0306 (5)	−0.0009 (3)	0.0026 (3)	−0.0145 (4)
N1	0.0116 (4)	0.0138 (4)	0.0191 (5)	−0.0003 (3)	0.0031 (3)	−0.0059 (3)
N2	0.0135 (4)	0.0134 (4)	0.0156 (4)	−0.0002 (3)	0.0024 (3)	−0.0038 (3)
C10	0.0131 (4)	0.0133 (4)	0.0132 (4)	0.0003 (3)	0.0026 (3)	−0.0025 (4)
C11	0.0136 (4)	0.0126 (4)	0.0129 (4)	0.0001 (3)	0.0026 (3)	−0.0021 (3)
C12	0.0145 (4)	0.0143 (5)	0.0164 (5)	0.0021 (3)	0.0000 (4)	−0.0032 (4)
C13	0.0165 (4)	0.0133 (4)	0.0171 (5)	0.0019 (4)	0.0012 (4)	−0.0031 (4)
C14	0.0159 (4)	0.0130 (4)	0.0140 (5)	−0.0026 (3)	0.0036 (4)	−0.0029 (4)
Br1	0.01679 (5)	0.01735 (6)	0.02281 (7)	−0.00640 (4)	0.00638 (4)	−0.00819 (5)
C15	0.0133 (4)	0.0176 (5)	0.0228 (6)	−0.0006 (4)	0.0006 (4)	−0.0050 (4)
C16	0.0134 (4)	0.0151 (5)	0.0225 (6)	0.0013 (4)	0.0015 (4)	−0.0053 (4)
C17	0.0126 (4)	0.0186 (5)	0.0187 (5)	−0.0004 (4)	0.0027 (4)	−0.0068 (4)

### 2-(Benzo[d]thiazol-2-yl)-N'-[(E)-1-(4-bromophenyl)ethylidene]acetohydrazide Geometric parameters (Å, º)

**Table:**

S1—C7A	1.7342 (12)	C12—C13	1.3950 (17)
S1—C2	1.7507 (12)	C13—C14	1.3862 (17)
C2—N3	1.2976 (16)	C14—C15	1.3919 (18)
C2—C8	1.4977 (17)	C14—Br1	1.8975 (12)
N3—C3A	1.3909 (16)	C15—C16	1.3905 (18)
C3A—C4	1.3998 (17)	C4—H4	0.9500
C3A—C7A	1.4090 (17)	C5—H5	0.9500
C4—C5	1.385 (2)	C6—H6	0.9500
C5—C6	1.405 (2)	C7—H7	0.9500
C6—C7	1.3875 (19)	C8—H8A	0.9900
C7—C7A	1.3973 (17)	C8—H8B	0.9900
C8—C9	1.5144 (16)	N1—H01	0.91 (2)
C9—O1	1.2307 (15)	C12—H13	0.9500
C9—N1	1.3543 (15)	C13—H14	0.9500
N1—N2	1.3764 (15)	C15—H16	0.9500
N2—C10	1.2942 (15)	C16—H17	0.9500
C10—C11	1.4849 (17)	C17—H11A	0.9800
C10—C17	1.5044 (17)	C17—H11B	0.9800
C11—C12	1.3999 (17)	C17—H11C	0.9800
C11—C16	1.4057 (17)		
			
C7A—S1—C2	89.24 (6)	C16—C15—C14	118.95 (11)
N3—C2—C8	122.18 (11)	C15—C16—C11	121.23 (11)
N3—C2—S1	115.90 (9)	C5—C4—H4	120.7
C8—C2—S1	121.90 (9)	C3A—C4—H4	120.7
C2—N3—C3A	110.63 (10)	C4—C5—H5	119.5
N3—C3A—C4	125.01 (11)	C6—C5—H5	119.5
N3—C3A—C7A	115.16 (10)	C7—C6—H6	119.3
C4—C3A—C7A	119.84 (11)	C5—C6—H6	119.3
C5—C4—C3A	118.66 (12)	C6—C7—H7	121.2
C4—C5—C6	120.91 (12)	C7A—C7—H7	121.2
C7—C6—C5	121.40 (12)	C2—C8—H8A	108.9
C6—C7—C7A	117.51 (12)	C9—C8—H8A	108.9
C7—C7A—C3A	121.67 (11)	C2—C8—H8B	108.9
C7—C7A—S1	129.27 (10)	C9—C8—H8B	108.9
C3A—C7A—S1	109.07 (8)	H8A—C8—H8B	107.8
C2—C8—C9	113.15 (10)	C9—N1—H01	116.1 (15)
O1—C9—N1	120.57 (11)	N2—N1—H01	124.4 (15)
O1—C9—C8	122.41 (11)	C13—C12—H13	119.5
N1—C9—C8	117.01 (10)	C11—C12—H13	119.5
C9—N1—N2	119.49 (10)	C14—C13—H14	120.4
C10—N2—N1	116.53 (10)	C12—C13—H14	120.4
N2—C10—C11	115.41 (10)	C16—C15—H16	120.5
N2—C10—C17	123.54 (11)	C14—C15—H16	120.5
C11—C10—C17	121.04 (10)	C15—C16—H17	119.4
C12—C11—C16	118.30 (11)	C11—C16—H17	119.4
C12—C11—C10	121.47 (10)	C10—C17—H11A	109.5
C16—C11—C10	120.23 (10)	C10—C17—H11B	109.5
C13—C12—C11	120.98 (11)	H11A—C17—H11B	109.5
C14—C13—C12	119.25 (11)	C10—C17—H11C	109.5
C13—C14—C15	121.28 (11)	H11A—C17—H11C	109.5
C13—C14—Br1	119.58 (9)	H11B—C17—H11C	109.5
C15—C14—Br1	119.09 (9)		
			
C7A—S1—C2—N3	−0.02 (10)	C2—C8—C9—O1	−10.43 (19)
C7A—S1—C2—C8	178.31 (10)	C2—C8—C9—N1	170.32 (12)
C8—C2—N3—C3A	−178.76 (11)	O1—C9—N1—N2	178.97 (12)
S1—C2—N3—C3A	−0.44 (13)	C8—C9—N1—N2	−1.76 (18)
C2—N3—C3A—C4	−178.97 (12)	C9—N1—N2—C10	−179.12 (12)
C2—N3—C3A—C7A	0.82 (14)	N1—N2—C10—C11	179.74 (11)
N3—C3A—C4—C5	−179.62 (12)	N1—N2—C10—C17	−1.04 (19)
C7A—C3A—C4—C5	0.60 (18)	N2—C10—C11—C12	−160.60 (12)
C3A—C4—C5—C6	0.1 (2)	C17—C10—C11—C12	20.16 (18)
C4—C5—C6—C7	−0.7 (2)	N2—C10—C11—C16	19.43 (18)
C5—C6—C7—C7A	0.57 (19)	C17—C10—C11—C16	−159.82 (12)
C6—C7—C7A—C3A	0.15 (18)	C16—C11—C12—C13	0.41 (19)
C6—C7—C7A—S1	−179.52 (10)	C10—C11—C12—C13	−179.56 (12)
N3—C3A—C7A—C7	179.45 (11)	C11—C12—C13—C14	0.6 (2)
C4—C3A—C7A—C7	−0.75 (18)	C12—C13—C14—C15	−0.8 (2)
N3—C3A—C7A—S1	−0.82 (13)	C12—C13—C14—Br1	176.63 (10)
C4—C3A—C7A—S1	178.98 (9)	C13—C14—C15—C16	0.1 (2)
C2—S1—C7A—C7	−179.84 (12)	Br1—C14—C15—C16	−177.39 (11)
C2—S1—C7A—C3A	0.46 (9)	C14—C15—C16—C11	1.0 (2)
N3—C2—C8—C9	−115.73 (13)	C12—C11—C16—C15	−1.2 (2)
S1—C2—C8—C9	66.05 (14)	C10—C11—C16—C15	178.79 (13)

### 2-(Benzo[d]thiazol-2-yl)-N'-[(E)-1-(4-bromophenyl)ethylidene]acetohydrazide Hydrogen-bond geometry (Å, º)

**Table:**

*D*—H···*A*	*D*—H	H···*A*	*D*···*A*	*D*—H···*A*
N1—H01···O1^i^	0.91 (2)	1.98 (2)	2.8839 (14)	168 (2)
C7—H7···N3^ii^	0.95	2.68	3.5942 (17)	161
C17—H11*B*···Br1^iii^	0.98	2.93	3.7666 (12)	145
C17—H11*C*···N2^iv^	0.98	2.69	3.5464 (18)	147

Symmetry codes: (i) −*x*+1, −*y*+2, −*z*+1; (ii) *x*, *y*+1, *z*; (iii) −*x*+1, *y*+1/2, −*z*+1/2; (iv) −*x*+1, −*y*+1, −*z*+1.
